# Prospective Validation of Candidate SNPs of *VEGF/VEGFR* Pathway in Metastatic Colorectal Cancer Patients Treated with First-Line FOLFIRI Plus Bevacizumab

**DOI:** 10.1371/journal.pone.0066774

**Published:** 2013-07-04

**Authors:** Fotios Loupakis, Chiara Cremolini, Dongyun Yang, Lisa Salvatore, Wu Zhang, Takeru Wakatsuki, Pierre Bohanes, Marta Schirripa, Leonor Benhaim, Sara Lonardi, Carlotta Antoniotti, Giuseppe Aprile, Francesco Graziano, Annamaria Ruzzo, Sara Lucchesi, Monica Ronzoni, Ferdinando De Vita, Giuseppe Tonini, Alfredo Falcone, Heinz-Josef Lenz

**Affiliations:** 1 University of Southern California Norris Comprehensive Cancer Center, Los Angeles, California, United States of America; 2 Polo Oncologico, Azienda Ospedaliero-Universitaria Pisana, Istituto Toscano Tumori, Pisa, Italy; 3 Unit of Medical Oncology 1, Oncology Institute of Veneto, Padua, Italy; 4 Azienda Ospedaliero-Universitaria di Udine, Udine, Italy; 5 Unit of Medical Oncology, Azienda Ospedaliera Ospedale San Salvatore, Pesaro, Italy; 6 Section of Biochemistry and Molecular Biology “G. Fornaini”, Department of Biomolecular Sciences, University of Urbino, Urbino, Italy; 7 Unit of Medical Oncology, Azienda USL5 Pontedera, Istituto Toscano Tumori, Pisa, Italy; 8 Unit of Oncology, Istituto Scientifico San Raffaele, Milan, Italy; 9 Unit of Medical Oncology, Seconda Università di Napoli, Naples, Italy; 10 Unit of Medical Oncology, University Campus Biomedico, Rome, Italy; MOE Key Laboratory of Environment and Health, School of Public Health, Tongji Medical College, Huazhong University of Science and Technology, China

## Abstract

**Purpose:**

The potential impact of different SNPs of *VEGF/VEGFR* pathway on the clinical outcome of mCRC patients receiving bev-containing regimens has been investigated in retrospective experiences with contrasting results. We previously reported the association of *VEGFA* rs833061 C/T variants with PFS in metastatic colorectal cancer patients treated with first-line FOLFIRI plus bevacizumab. The primary objective of this work was to prospectively validate that retrospective finding. A confirmatory analysis of other SNPs of *VEGF/VEGFR* pathway genes was included.

**Experimental design:**

To detect a HR for PFS of 1.7 for *VEGFA* rs833061 T/T compared to C- variants in metastatic colorectal cancer patients treated with first-line FOLFIRI plus bevacizumab, setting two-sided α = 0.05 and β = 0.20, 199 events were required. *VEGFA* rs699946 A/G, rs699947 A/C, *VEGFR1* rs9582036 A/C and rs7993418 A/G, *VEGFR2* rs11133360 C/T, rs12505758 C/T and rs2305948 C/T and *EPAS1* rs4145836 A/G were also tested. Germ-line DNA was extracted from peripheral blood. SNPs were analyzed by PCR and sequencing.

**Results:**

Four-hundred-twenty-four pts were included. At the univariate analysis, no differences according to *VEGFA* rs833061 C/T variants were observed in PFS (p = 0.38) or OS (p = 0.95). Among analyzed SNPs, only *VEGFR2* rs12505758 C- variants, compared to T/T, were associated to shorter PFS (HR: 1.36 [1.05–1.75], p = 0.015, dominant genetic model) and OS, with a trend toward significance (HR: 1.34 [0.95–1.88], p = 0.088). In the multivariate model, this association retained significance (HR: 1.405 [1.082–1.825], p = 0.012) in PFS, that was lost by applying multiple testing correction (p = 0.14).

**Conclusion:**

This prospective experience failed to validate the hypothesized predictive impact of *VEGFA* rs833061 variants. Retrospective findings on different candidate SNPs were not confirmed. Only *VEGFR2* rs12505758 variants, whose prognostic and not predictive impact was previously reported, correlated with PFS. Given the complexity of angiogenesis, it is rather unlike that a single germ-line SNP might be a good predictor of benefit from bevacizumab.

## Introduction

The inhibition of angiogenesis, through the blockade of VEGF/VEGFR pathway, is an effective strategy in the treatment of metastatic colorectal cancer (mCRC). Not only the anti-VEGFA monoclonal antibody bevacizumab (BV) [1], [2] but also, in the very last months, the VEGF and PlGF trap aflibercept [3] and the multikinase inhibitor regorafenib [4] have demonstrated significant advantages in terms of survival.

By a clinical perspective, the relatively small absolute benefit provided by these new agents, as well as the availability of an increasing number of therapeutic options make the identification of predictive biomarkers an essential need in order to optimize the use of antiangiogenic agents. Unfortunately, up today, this need is still unsolved.

With regard to BV, despite several attempts spanning from the pharmacodynamic to the imaging approach [5]–[6], no biomarkers of benefit or resistance have been identified so far. Looking at the biology of angiogenesis, increasing evidences highlight the contribution of tumour microenvironment to cancer progression. The so called “niche” [7]–[8], including endothelial and mesenchimal cells, plays a central role in the growth of new vessels, that is therefore a largely host-mediated, besides tumor-mediated, phenomenon. Based on these considerations, the pharmacogenetic approach, evaluating the impact of germ-line variability on drugs’ efficacy, earned success as a promising tool to disclose potential predictors of benefit. Nevertheless, up today, several retrospective experiences have provided inconclusive and even contrasting results [9]–[14].

Our group retrospectively investigated the association of 4 *VEGFA* SNPs with survival parameters in a cohort of 111 mCRC patients treated with upfront FOLFIRI plus BV and in a historical, non-randomized, cohort of 107 mCRC patients treated with upfront FOLFIRI alone [10]. A significant association of *VEGFA* rs833061 C/T allelic variants with PFS and OS was reported in the BV-group, but not in the control group. When treated with BV, patients bearing *VEGFA* rs833061 T/T genotype had significantly shorter PFS compared to those carrying at least one C- allele, both in the univariate and in the multivariate model. Moreover, the significance of an exploratory interaction test, though affected by the non-randomization bias, suggested that this association could be actually related to the effect of BV.

Our goal was to validate prospectively the association of this SNP with outcome in a clinical trial designed and powered to confirm the SNP as a predictive biomarker in a population of previously untreated mCRC receiving first-line FOLFIRI plus BV, just like the population included in the retrospective cohort.

In the meanwhile, new appealing results were provided by the largest pharmacogenetic analysis related to BV and the outcome of patients with different solid malignancies, randomized to receive or not the antiangiogenic drug in first-line randomized phase III trials [15]. Among 158 investigated SNPs in potentially relevant genes, Lambrechts et al. identified some promising SNPs in *VEGFA, VEGFR1/2* and *EPAS1*. We therefore included as secondary endpoints of our prospective trial the evaluation of all those SNPs that showed a possible correlation with the outcome in the retrospective study presented by Lambrechts et al.

Here we present the first prospective evaluation of candidate SNPs of VEGF/VEGFR pathway as potential predictors of clinical outcome in a large and clinically homogenous cohort of mCRC patients treated with first-line FOLFIRI plus BV. Currently available evidences about the potential predictive and/or prognostic power of investigated SNPs are summarized in Table 1.

**Table 1:** Investigated SNPs: current evidences from literature.

Gene	rs number	Major/Minor allele	Major findings
*VEGFA*	833061	C/T	• Associated with PFS and OS in a retrospective cohort of mCRC patients treated with first-line FOLFIRI+BV
			• Not associated with clinical outcome in CAPOX+BV arm of CAIRO-2 trial in mCRC
*VEGFA*	699946	A/G	• Predictive for PFS in patients treated with or without BV in phase III randomized trials.
*VEGFA*	699947	C/A	• Predictive for OS in E2100 trial of paclitaxel±BV in metastatic breast cancer.
			• Not associated with clinical outcome in CAPOX+BV arm of CAIRO-2 trial or in in a retrospective analysis of patients treated with or without BV in phase III randomized trials.
*VEGFR1*	7993418	A/G	• Predictive for PFS in AVOREN trial of IFN-a±BV in metastatic renal cancer
*VEGFR1*	9582036	A/C	• Predictive for PFS and OS in AViTA trial of gemcitabine+erlotinib±BV in metastatic pancreatic adenocarcinoma
			• Predictive for PFS in AVOREN trial of IFN-a±BV in metastatic renal cancer
*VEGFR2*	11133360	T/C	• Associated with PFS in patients treated with BV in phase III randomized trials
*VEGFR2*	12505758	T/C	• Associated with OS in patients treated with BV in phase III randomized trials
*VEGFR2*	2305948	C/T	• Associated with response in a retrospective cohort of mCRC patients treated with oxaliplatin-containing chemotherapy+BV
*EPAS1*	4145836	G/A	• Associated with PFS in patients treated with, but also without BV, in phase III randomized trials

## Patients and Methods

### Eligibility Criteria and Study Procedures

Patients with histologically confirmed diagnosis of metastatic colorectal adenocarcinoma were enrolled in the trial if they were more than 18 years old, had at least one measurable lesion according to RECIST 1.0 and had never been treated for metastatic disease. Previous adjuvant oxaliplatin was allowed if more than 12 months had elapsed between the end of adjuvant therapy and relapse. Adequate bone marrow, liver and renal function were required. All involved subjects signed their written informed consent to study treatment and related procedures. The trial was approved by the local ethics committee (Comitato Etico Sperimentazione Farmaco - Azienda Ospedaliero-Universitaria Pisana) and clinical investigation was conducted according to the Declaration of Helsinki.

Study treatment consisted of biweekly administrations of BV 5 mg/kg ev at day 1, followed by Irinotecan 165 mg/sqm ev, infused concomitantly with L-Leucovorin 200 mg/sqm ev, followed by 5-fluorouracil 400 mg/sqm ev and 5-fluoruracil 2400 mg/sqm as a 48-h continuous infusion starting on day 1. Irinotecan was administered for a maximum of 12 cycles or until progressive disease, unacceptable toxicities or patients’ refusal. 5-fluorouracil, L-Leucovorin and BV were continued until the evidence of progressive disease, unacceptable toxicities or patients’ refusal.

Response was assessed by means of CT scan, that was repeated every 8 weeks. RECIST criteria v1.0 were applied.

Six ml blood samples were collected in EDTA tubes and stored at −20°C.

### Statistical Design

Based on our previous retrospective findings, we designed a prospective phase II trial. According to Schoenfeld design, in order to detect a negative effect of *VEGFA rs833061* T/T variant compared to C- variants equal to a HR for PFS of 1.7, adopting two-sided α = 0.05 and β = 0.20 respectively, and assuming a prevalence of T/T variant of 25%, we estimated to require 199 events.

The association of polymorphisms with PFS and OS was analyzed using Kaplan–Meier curves and the log-rank test.

PFS was defined as the time from the first administration of study treatment until the first documentation of objective disease progression according to RECIST 1.0, or death due to any cause, whichever occurs first. Patients undergoing secondary radical resection of metastatic lesions were censored at the time of surgery.

OS was defined as the time from the first administration of study treatment until the date of death due to any cause.

The associations between polymorphisms and RR were examined using contingency tables and the Cochran-Mantel-Haenszel test.

Since 9 SNPs were tested, the Benjamini and Hochberg method was used to control the false discovery rate (FDR) of multiple hypothesis testing. Polymorphisms that were significantly associated with clinical outcome with an FDR-adjusted P value of <0.15 were chosen to include in the multivariate model.

In the multivariate Cox regression analysis for PFS and OS, models were adjusted for mucinous histology, ECOG PS, baseline LDH level, number of metastatic sites and primary tumour site. We used the stepwise Cox regression model to choose covariates that were accounted for in the multivariate model. Covariates that remained significant at a 0.1 level in the multivariate analysis were retained.

When applying multiple testing correction for the number of analyzed SNPs, the association of *VEGFR2* 12505758 C/T variants with clinical outcome was not significant (p = 0.14).

All analyses were carried out using the SAS statistical package version 9.2.

This study has been completed and is registered in ClinicalTrials.gov, with number NCT01363739. Study treatment was not issued for the purpose of this trial; patients would have received the same regimen independently of their enrollment.

### Genotyping

Genomic DNA was extracted from peripheral blood using the QIAamp Kit (Qiagen). *VEGFA* rs833061 C/T, rs699946 A/G and rs699947 A/C, *VEGFR1* rs9582036 A/C and rs7993418 A/G, *VEGFR2* rs11133360 C/T, rs12505758 C/T and rs2305948 C/T and *EPAS1* rs4145836 A/G SNPs were gentyped by means of PCR and sequencing. Investigators conducting genetic analyses were blinded to patients’ characteristics and clinical outcome.

## Results

From April 2006 to May 2011, 424 mCRC patients have been enrolled in 35 Italian Oncology Units. Main patients’ characteristics and correlations with clinical outcome are summarized in Table 2 and 3.

**Table 2:** Baseline characteristics and RECIST response.

		Tumor Response				
	*N*	CR	PR	SD	PD	*P* * value
Age, years						0.95
≤65	274	16 (6%)	145 (54%)	77 (29%)	31 (12%)	
>65	150	9 (6%)	74 (50%)	54 (36%)	11 (7%)	
Sex						0.040
M	252	12 (5%)	126 (51%)	81 (33%)	30 (12%)	
F	172	13 (8%)	93 (55%)	50 (30%)	12 (7%)	
ECOGPS						0.003
0	357	21 (6%)	194 (55%)	111 (31%)	27 (8%)	
1–2	67	4 (6%)	25 (39%)	20 (31%)	15 (23%)	
Primary tumor site						0.11
Right colon	107	7 (7%)	49 (48%)	32 (31%)	15 (15%)	
Left colon	180	13 (7%)	100 (56%)	55 (31%)	10 (6%)	
Rectum	122	5 (4%)	67 (55%)	38 (31%)	12 (10%)	
Colon, rectum	1					
Unknown	14					
Mucinous histology						<.001
Yes	52	1 (2%)	18 (36%)	25 (50%)	6 (12%)	
No	261	23 (9%)	149 (57%)	66 (25%)	23 (9%)	
NA	111	1 (1%)	52 (49%)	40 (38%)	13 (12%)	
Liver-only disease						0.032
Yes	136	11 (8%)	77 (57%)	37 (27%)	10 (7%)	
No	288	14 (5%)	142 (50%)	94 (33%)	32 (11%)	
Mst site, n						0.016
1	194	19 (10%)	100 (52%)	58 (30%)	15 (8%)	
>1	230	6 (3%)	119 (53%)	73 (32%)	27 (12%)	
Time to mets						0.34
Synchronous	311	17 (6%)	169 (56%)	87 (29%)	31 (10%)	
Metachronous	113	8 (7%)	50 (44%)	44 (39%)	11 (10%)	
Kohne Score						0.012
Low	194	19 (10%)	101 (53%)	58 (30%)	14 (7%)	
Intermediate	180	6 (3%)	97 (55%)	53 (30%)	20 (11%)	
High	35	0 (0%)	14 (41%)	15 (44%)	5 (15%)	
NA	15	0 (0%)	7 (47%)	5 (33%)	3 (20%)	
Primary tumorresected						0.86
Yes	320	21 (7%)	159 (50%)	107 (34%)	29 (9%)	
No	103	4 (4%)	60 (60%)	24 (24%)	12 (12%)	
Unknown	1					
High LDH						0.077
Yes	188	10 (5%)	104 (56%)	52 (28%)	19 (10%)	
No	188	15 (8%)	93 (51%)	60 (33%)	16 (9%)	
NA	48	0 (0%)	22 (46%)	19 (40%)	7 (15%)	
Prior adjuvant CT						
No	333	19 (6%)	183 (56%)	93 (28%)	32 (10%)	0.083
Yes	91	6 (7%)	36 (40%)	38 (42%)	10 (11%)	

* *P* value was based on Cochran-Mantel-Haenszel test for response and log-rank test for PFS and OS.

**Table 3:** Baseline characteristics and survival outcomes.

		Progression-Free Survival			Overall Survival		
	*N*	Median PFS,mos (95%CI)	HR (95%CI)	*P* * value	Median OS,mos (95%CI)	HR (95%CI)	*P* * value
Age, years				0.22			0.78
≤65	274	10.2 (9.5, 10.8)	1 (Reference)		29.6 (23.9, 37.8)	1 (Reference)	
>65	150	11.8 (10.0, 13.5)	0.86 (0.68, 1.09)		31.2 (23.8, 42.0)	0.96 (0.70, 1.31)	
Sex				0.49			0.69
M	252	10.3 (9.5, 11.4)	1 (Reference)		26.3 (22.9, 37.8)	1 (Reference)	
F	172	10.8 (9.6, 11.7)	0.92 (0.73, 1.16)		31.2 (27.3, 44.1)	0.94 (0.69, 1.28)	
ECOGPS				<.001			<.001
0	357	10.9 (10.2, 11.8)	1 (Reference)		33.1 (29.8, 42.0)	1 (Reference)	
1–2	67	7.9 (6.3, 10.0)	1.71 (1.27, 2.28)		18.6 (13.9, 21.4)	2.74 (1.90, 3.95)	
Primarytumor site				0.002			<.001
Right colon	107	9.7 (8.8, 10.3)	1 (Reference)		23.5 (19.1, 27.3)	1 (Reference)	
Left colon	180	11.5 (10.5, 14.0)	0.61 (0.46, 0.81)		42.0 (28.6, 53.5)	0.48 (0.33, 0.69)	
Rectum	122	10.6 (8.7, 11.8)	0.80 (0.60, 1.09)		32.6 (25.4, 48.5)	0.56 (0.38, 0.82)	
Colon, rectum	1						
Unknown	14						
Mucinous histology				<.001			0.16
Yes	52	9.6 (8.4, 11.2)	1 (Reference)		25.4 (19.9, 42.0)	1 (Reference)	
No	261	11.3 (10.4, 12.4)	0.76 (0.54, 1.06)		32.6 (28.6, 38.0)	0.69 (0.45, 1.06)	
NA	111	8.3 (7.8, 9.5)	1.36 (0.89, 2.06)		24.8 (20.0, 40.3)	0.89 (0.50, 1.59)	
Liver-only disease				0.028			0.14
Yes	136	11.5 (9.8, 12.6)	1 (Reference)		33.0 (23.7, 53.5)	1 (Reference)	
No	288	10.2 (9.6, 11.1)	1.34 (1.03, 1.75)		29.2 (23.8, 37.8)	1.29 (0.91, 1.81)	
Mst site, n				<.001			<.001
1	194	11.9 (10.5, 13.7)	1 (Reference)		34.6 (29.6, 53.5)	1 (Reference)	
>1	230	9.8 (9.3, 10.5)	1.61 (1.27, 2.05)		24.8 (21.6, 31.2)	1.79 (1.30, 2.48)	
Time to mets				0.94			0.066
Synchronous	311	10.3 (9.6, 11.3)	1 (Reference)		26.3 (23.5, 32.1)	1 (Reference)	
Metachronous	113	10.5 (9.5, 13.5)	0.99 (0.76, 1.28)		38.0 (29.6, 49.2)	0.72 (0.50, 1.03)	
Kohne Score				<.001			<.001
Low	194	11.9 (10.5, 13.5)	1 (Reference)		34.6 (29.8, 53.5)	1 (Reference)	
Intermediate	180	10.2 (9.6, 11.3)	1.50 (1.16, 1.93)		29.1 (23.2, 37.8)	1.59 (1.12, 2.25)	
High	35	7.9 (6.6, 8.6)	2.47 (1.64, 3.72)		18.9 (10.2, 21.4)	3.67 (2.26, 5.96)	
NA	15	9.8 (7.3, 12.2)	1.73 (1.00, 2.98)		31.2 (6.9, 39.3)	1.79 (0.88, 3.64)	
Primary tumor resected				0.022			0.008
Yes	320	10.8 (10.1, 11.6)	1 (Reference)		31.6 (27.3, 44.1)	1 (Reference)	
No	103	9.5 (8.2, 11.0)	1.37 (1.04, 1.81)		23.5 (19.1, 32.6)	1.62 (1.12, 2.33)	
Unknown	1						
High LDH				0.016			0.35
Yes	188	10.2 (9.3, 11.3)	1 (Reference)		28.6 (21.8, 42.0)	1 (Reference)	
No	188	11.1 (10.1, 13.1)	0.77 (0.60, 0.98)		31.6 (24.8, 38.4)	0.83 (0.60, 1.16)	
NA	48	9.5 (8.1, 11.0)	1.23 (0.85, 1.79)		31.2 (19.6, 49.2)	1.14 (0.69, 1.87)	
Prior adjuvant CT							
No	333	10.4 (9.6, 11.4)	1 (Reference)	0.90	28.8 (23.8, 33.0)	1 (Reference)	0.28
Yes	91	10.5 (9.6, 13.7)	1.02 (0.77, 1.34)		38.0 (25.9, 53.5)	0.82 (0.56, 1.19)	

* *P* value was based on Cochran-Mantel-Haenszel test for response and log-rank test for PFS and OS.

At a median follow up of 24 months, 292 patients were progressed and 164 died. In the overall population median PFS and OS were 10.5 and 29.9 months, respectively. Two-hundred-nineteen (53%) and 25 (6%) out of 417 evaluated patients showed partial or compete response. One-hundred-thirty-one (31%) patients showed disease stabilization, while 42 (10%) patients progressed. The objective response rate was 59% and the disease control rate was 90%. Forty-six (11%) patients underwent secondary R0 resection of metastatic lesions.

### Primary Endpoint

**Figure 1:**
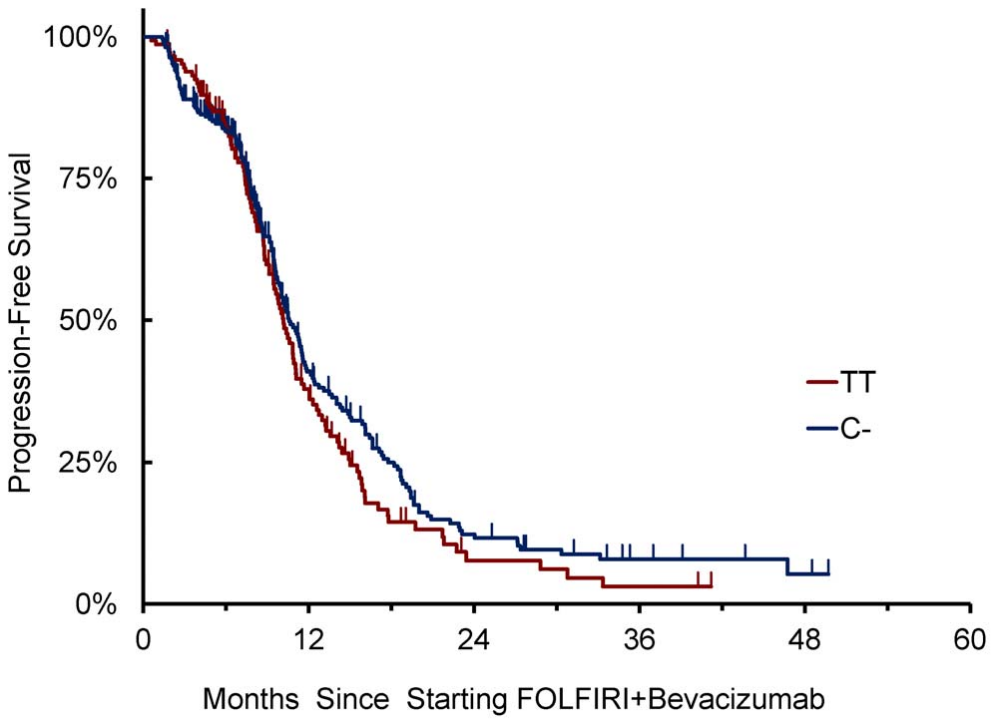
*VEGFA 833061* C/T allelic variants and PFS.

**Figure 2:**
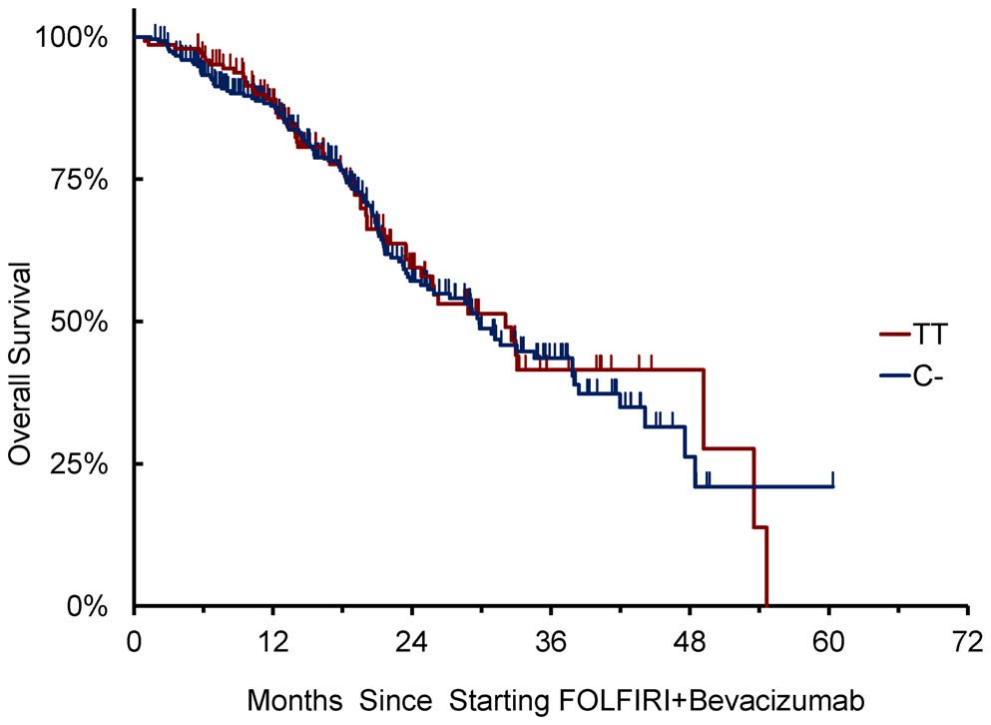
*VEGFA 833061* C/T allelic variants and OS.

*VEGFA* rs833061 C/T SNP was successfully genotyped in 423 cases. No association of *VEGFA* rs833061 C/T variants with clinical outcome was observed (Table 4 and 5). Patients homozygous for T/T (N = 147) showed a median PFS of 10.2 months vs 10.0 months for patients bearing at least one C- allele (N = 276) (HR: 1.17 [95%CI: 0.91–1.50], p = 0.218) (Figure 1). Also in terms of OS, patients homozygous for T/T showed a median OS of 32.1 months vs 29.9 months for patients bearing at least one C- allele (HR: 0.96 [95%CI: 0.70–1.33], p = 0.733) (Figure 2).

**Table 4:** *VEGFA 833061* SNP allelic variants and survival.

		Progression-Free Survival			Overall Survival		
	*N*	Median PFS, mos (95%CI)	HR (95%CI)	*P* * value	Median OS, mos (95%CI)	HR (95%CI)	*P* * value
***VEGFA*** ** 833061**							
**TT**	147	10.2 (9.1, 11.1)	1 (Reference)		32.1 (23.8, 53.5)	1 (Reference)	
**CT**	197	10.6 (9.6, 11.9)	0.85 (0.66, 1.09)	0.38	30.9 (23.7, 38.4)	1.05 (0.74, 1.49)	0.95
**CC**	79	10.4 (9.4, 12.7)	0.85 (0.61, 1.17)		29.8 (21.6, 44.1)	1.00 (0.64, 1.55)	

* *P* value was based on Cochran-Mantel-Haenszel test for response and log-rank test for PFS and OS.

**Table 5:** *VEGFA 833061* SNP allelic variants and response.

		Tumor Response				
	*N*	CR	PR	SD	PD	*P* * value
***VEGFA*** ** 833061**						
**TT**	147	4 (3%)	83 (57%)	48 (33%)	11 (8%)	
**CT**	197	14 (7%)	96 (50%)	61 (32%)	21 (11%)	0.99
**CC**	79	7 (9%)	39 (50%)	22 (28%)	10 (13%)	

* *P* value was based on Cochran-Mantel-Haenszel test for response and log-rank test for PFS and OS.

### Secondary Endpoints

As reported in Table 6 and 7, no significant association of *VEGFA* rs699946 A/G and rs699947 A/C, *VEGFR1* rs9582036 A/C and rs7993418 A/G, *VEGFR2* rs11133360 C/T and rs2305948 C/T and *EPAS1* rs4145836 A/G allelic variants with clinical outcome was observed.

**Table 6:** Other candidate *VEGFA*, *VEGFR1*, *VEGFR2* and *EPAS1* SNPs allelic variants and RECIST response.

		Tumor Response				
	*N*	CR	PR	SD	PD	*P* * value
***VEGFA*** ** 699946**						
**AA**	257	17 (7%)	127 (50%)	80 (32%)	29 (11%)	
**AG**	144	8 (6%)	77 (55%)	47 (33%)	9 (6%)	0.59
**GG**	23	0 (0%)	15 (65%)	4 (17%)	4 (17%)	
***VEGF*** **A 699947**						
**CC**	148	4 (3%)	84 (57%)	49 (33%)	10 (7%)	
**AC**	199	14 (7%)	97 (50%)	61 (31%)	22 (11%)	0.94
**AA**	77	7 (9%)	38 (50%)	21 (28%)	10 (13%)	
***VEGFR1*** ** 7993418**						
**AA**	270	12 (5%)	142 (54%)	87 (33%)	24 (9%)	
**AG**	138	12 (9%)	68 (50%)	39 (29%)	17 (13%)	0.89
**GG**	16	1 (6%)	9 (56%)	5 (31%)	1 (6%)	
***VEGFR1*** ** 9582036**						
**AA**	241	11 (5%)	128 (54%)	75 (32%)	23 (10%)	
**AC**	158	11 (7%)	80 (51%)	47 (30%)	18 (12%)	0.72
**CC**	25	3 (13%)	11 (46%)	9 (38%)	1 (4%)	
***VEGFR2*** ** 11133360**						
**TT**	143	9 (6%)	81 (57%)	38 (27%)	13 (9%)	
**CT**	212	13 (6%)	103 (50%)	69 (33%)	23 (11%)	0.45
**CC**	69	3 (4%)	35 (51%)	24 (35%)	6 (9%)	
***VEGFR2*** ** 12505758**						
**TT**	306	22 (7%)	156 (52%)	95 (31%)	29 (10%)	
**CT**	107	3 (3%)	55 (53%)	34 (33%)	12 (12%)	0.50
**CC**	11	0 (0%)	8 (73%)	2 (18%)	1 (9%)	
***VEGFR2*** ** 2305948**						
**CC**	354	22 (6%)	186 (54%)	106 (31%)	33 (10%)	
**CT†**	66	3 (4%)	33 (47%)	25 (36%)	9 (13%)	0.16
**TT†**	4					
***EPAS1*** ** 4145836**						
**GG**	332	22 (7%)	175 (54%)	96 (29%)	33 (10%)	
**AG†**	87	3 (3%)	44 (48%)	35 (38%)	9 (10%)	0.18
**AA†**	5					

* *P* value was based on Cochran-Mantel-Haenszel test for response and log-rank test for PFS and OS.

**Table 7:** Other candidate *VEGFA*, *VEGFR1*, *VEGFR2* and *EPAS1* SNPs allelic variants and survival outcomes.

		Progression-Free Survival			Overall Survival		
	*N*	Median PFS,mos (95%CI)	HR (95%CI)	*P* * value	Median OS,mos (95%CI)	HR (95%CI)	*P* * value
***VEGFA*** ** 699946**							
**AA**	257	10.4 (9.8, 11.5)	1 (Reference)		29.1 (23.5, 37.8)	1 (Reference)	
**AG**	144	10.5 (9.6, 11.9)	1.03 (0.80, 1.32)	0.34	33.0 (23.9, 42.0)	0.97 (0.70, 1.35)	0.69
**GG**	23	9.6 (8.1, 11.0)	1.44 (0.87, 2.39)		32.1 (16.3, 49.2+)	1.30 (0.68, 2.50)	
***VEGF*** **A 699947**							
**CC**	148	10.2 (9.5, 11.1)	1 (Reference)		32.6 (24.8, 53.5)	1 (Reference)	
**AC**	199	10.7 (9.6, 11.8)	0.86 (0.66, 1.11)	0.46	30.9 (23.5, 38.4)	1.11 (0.78, 1.57)	
**AA**	77	10.3 (9.4, 12.7)	0.86 (0.62, 1.19)		29.2 (21.2, 37.8)	1.06 (0.68, 1.65)	0.84
***VEGFR1*** ** 7993418**							
**AA**	270	10.5 (9.6, 11.6)	1 (Reference)		31.6 (25.9, 38.0)	1 (Reference)	
**AG**	138	10.3 (9.6, 11.3)	1.15 (0.90, 1.47)	0.52	28.6 (21.7, 37.8)	1.15 (0.83, 1.58)	0.57
**GG**	16	9.2 (7.8, 16.6)	0.97 (0.51, 1.84)		60.4+ (20.0, 60.4+)	0.82 (0.35, 1.90)	
***VEGFR1*** ** 9582036**							
**AA**	241	11.0 (10.0, 12.5)	1 (Reference)		32.6 (26.3, 44.1)	1 (Reference)	
**AC**	158	10.1 (9.5, 10.8)	1.25 (0.98, 1.58)	0.19	28.6 (23.3, 34.6)	1.17 (0.85, 1.60)	
**CC**	25	10.5 (7.8, 14.0)	1.15 (0.68, 1.96)		37.8 (20.0, 60.4+)	0.99 (0.51, 1.92)	0.62
***VEGFR2*** ** 11133360**							
**TT**	143	10.5 (9.7, 11.5)	1 (Reference)		33.0 (23.5, 60.4+)	1 (Reference)	
**CT**	212	10.8 (9.5, 12.3)	0.92 (0.71, 1.20)	0.60	30.9 (25.4, 34.6)	1.17 (0.83, 1.66)	0.42
**CC**	69	10.0 (8.6, 11.9)	1.08 (0.77, 1.51)		28.6 (20.8, 48.5+)	1.35 (0.85, 2.14)	
***VEGFR2*** ** 12505758**							
**TT**	306	10.9 (10.1, 11.7)	1 (Reference)		32.1 (26.3, 38.4)	1 (Reference)	
**CT**	107	9.5 (8.8, 11.1)	1.34 (1.03, 1.74)	**0.045**	23.5 (19.8, 29.9)	1.41 (1.00, 1.99)	0.12
**CC**	11	10.7 (9.3, 11.2)	1.57 (0.82, 2.98)		42.9+ (21.7, 42.9+)	0.75 (0.24, 2.38)	
***VEGFR2*** ** 2305948**							
**CC**	354	10.4 (9.6, 11.1)	1 (Reference)		29.9 (25.4, 34.6)	1 (Reference)	
**CT** †	66	11.5 (9.8, 12.5)	0.91 (0.66, 1.26)	0.57	29.8 (21.5, 49.7+)	0.89 (0.58, 1.37)	0.60
**TT** †	4						
***EPAS1*** ** 4145836**							
**GG**	332	10.6 (9.9, 11.5)	1 (Reference)		31.6 (25.9, 37.8)	1 (Reference)	
**AG** †	87	10.1 (8.1, 11.5)	0.95 (0.71, 1.25)	0.69	23.5 (19.9, 49.7+)	1.15 (0.80, 1.65)	0.46
**AA** †	5						

* *P* value was based on Cochran-Mantel-Haenszel test for response and log-rank test for PFS and OS.

† Dominant model.

A significant association of *VEGFR2* 12505758 C/T variants with clinical outcome was found in terms of PFS (p = 0.045), but not RR (p = 0.50) or OS (p = 0.12) using co-dominant genetic model.

**Figure 3:**
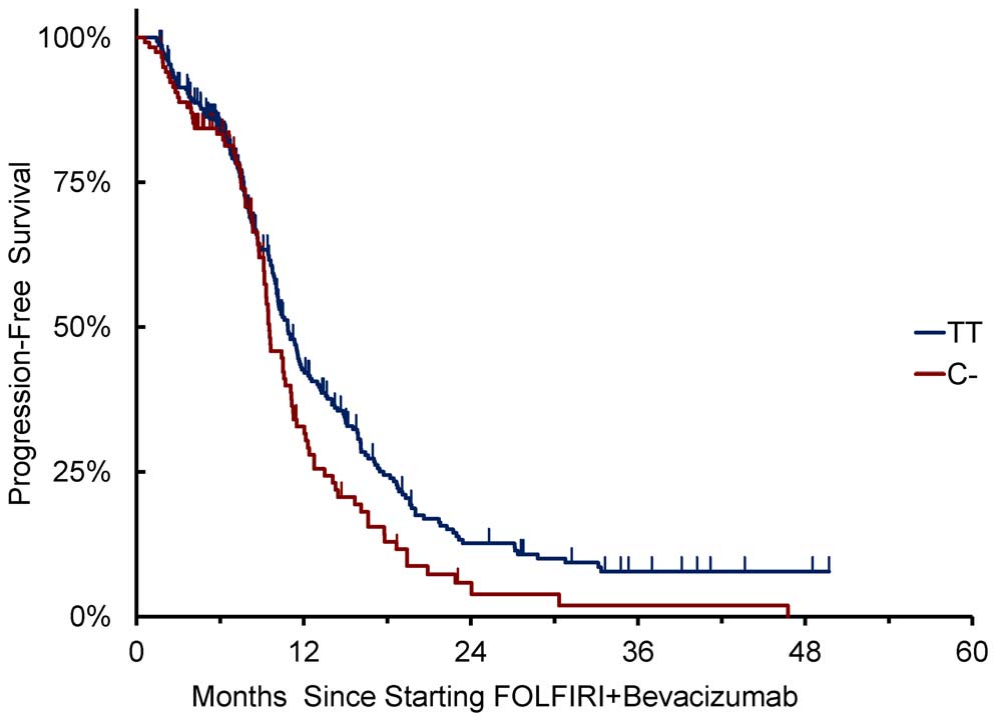
*VEGFR2 12505758* C/T allelic variants and PFS.

**Figure 4:**
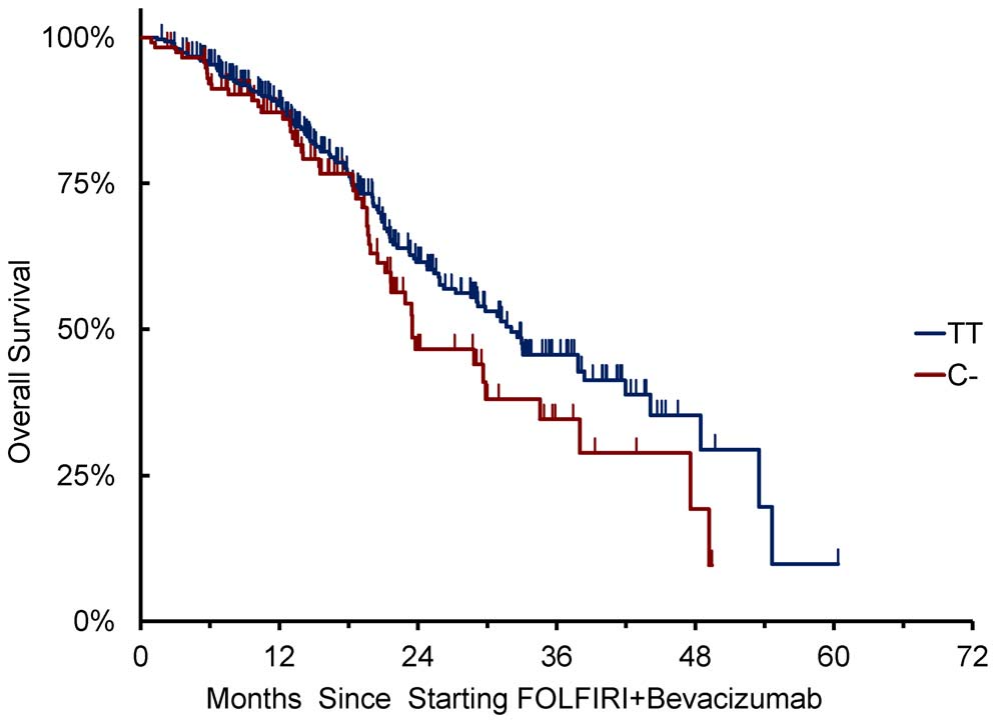
*VEGFR2 12505758* C/T allelic variants and OS.

Patients with at least one C- allele (N = 118) showed a median PFS of 9.5 months compared to 10.9 months of patients homozygous for T/T (N = 306) (HR: 1.36 [95%CI: 1.06–1.75], p = 0.015, dominant genetic model) (Figure 3). Patients with at least one C- allele showed a median OS of 23.5 months compared to 32.1 months of patients homozygous for T/T (N = 306) (HR: 1.34 [95%CI: 0.95–1.88], p = 0.088) (Figure 4). In the multivariate model, including mucinous histology, ECOG PS, baseline LDH levels, number of metastatic sites, and primary tumor site as covariates, *VEGFR2* 12505758 C- variants were still associated to shorter PFS compared to T/T variant (HR: 1.405 [1.082–1.825], p = 0.012). (Table S1 ).

## Discussion

In recent years, the search for biomarkers able to predict benefit from BV has been one of the most competitive translational research areas. Despite many efforts and some promising candidates, no markers have provided reliable and reproducible results, thus making this unsolved challenge extremely intriguing. The question remains why has it been so difficult to identify clinical relevant predictive markers for BV.

One of the most compelling reasons is the biologic complexity of tumor angiogenesis which is recognized as one of the hallmarks of cancer. In recent years data suggest the role of multiple pathways in the growth of new tumor vessels, leading to investigate the simultaneous inhibition of multiple angiogenic targets as a potentially efficacious strategy [16]. The role of several cell types in the development of such an intricate network of signals has also emerged and the contribution of both stromal cells and bone-marrow derived vascular progenitors, recruited and stimulated by hypoxic conditions, has been evidenced [17]. The relevance of tumor microenvironment as a crucial actor in new vessels’ growth and stabilization and the critical implication of the extracellular matrix in supporting neoangiogenesis are now well-established, thus confirming the contribution of both host- and tumor-related factors to the so called “angiogenic balance”.

All the mechanisms of action of anti-VEGFA therapy remain unclear since blockade of circulating VEGFA may impact not only tumors, but also stroma and endothelial cells’ proliferation and maturation. While BV antiangiogenic properties were firstly attributed to the inhibition of endothelial cells’ proliferation, today several different biologic effects are recognized, such as the inhibition of bone marrow-derived progenitors, the normalization of vessels’ structure, the vascular “constriction”, and the disruption of cancer stem cells’ niche, the direct effect on tumor cells and the interaction with the host immune system. As a consequence of these multiple mechanisms of action, the translation of *in vitro* and *in vivo* findings into the human model is not immediate and several difficulties make the development of efficacious preclinical models extremely complicated.

Another complicating factor in the identification of biomarkers of BV is that as a single agent has very limited antitumor effect, so that the confounding effect of chemo-backbone overshadows the BV effect. Moreover, non responding patients according to RECIST also achieve benefit in terms of prolonged time to tumor progression [18].

Despite these challenges, some potential candidates have emerged from different retrospective clinical trials. Although these markers were often considered worthy of further investigation, none of them was validated and entered clinical practice.

Our group recently showed the promising association of *VEGFA* rs833061C/T allelic variants with clinical outcome. However our biomarker was identified retrospectively in a clinical cohort, which was not randomized, and is biased by the multiple-testing approach [10].

This clinical trial represents our attempt to take up the challenge of validation, claimed by statisticians and methodology experts since the introduction of targeted drugs into clinical practice [19]. The need to validate retrospective findings, by conducting prospective trials is nowadays a matter of fact. Although randomized studies with an interaction design or a so-called “marker-based strategy” would provide the highest level of evidence, they would also require extremely large numbers of events [20]. A more pragmatic, but methodologically correct proposal is the “retrospective-prospective” approach, in which the hypothesis generated by the retrospective experience is prospectively challenged in a new cohort [21]. Up today, in spite of the high number of potentially interesting markers emerging from retrospective series, prospective trials with a formal statistical hypothesis have never been conducted. To the best of our knowledge, our work represents the first proof of concept sustaining this approach in the field of colorectal oncology.

The trial, designed on the basis of our retrospective findings, attests the failure of *VEGFA* rs833061C/T SNP as a potential predictor of benefit from BV and does not confirm previous results about other candidate SNPs.

With regard to *VEGFR2* 12505758 C/T SNP, whose prognostic rather than predictive impact has been previously suggested [16], we should acknowledge that the significance of the correlation with PFS was lost when applying the multiple testing correction. However, since VEGFR2 is acquiring growing relevance as a consequence of the affirmation of other targeted agents interfering with its function, like aflibercept and regorafenib, these results may deserve further investigation.

The encouraging result reported in terms of OS (median OS: 29.9 months) probably mirrors the accessibility of these patients, enrolled between April 2006 and May 2011, to all cytotoxics and targeted agents indicated for the treatment of mCRC as well as the improvement of locoregional techniques and the increasing expertise of committed surgeons.

In conclusion, though recognizing the relevance of preliminary retrospective experiences as essential starting points to generate new work-hypotheses, the present study confirms the absolute importance of the prospective validation as an essential step on biomarkers’ way toward clinical application.

Moreover, based on the complexity of tumor angiogenesis biology and the involvement of multiple actors in this process, it seems rather unlike that a single germ-line SNP might account for the efficacy of BV by itself. The failure of the “candidate SNP strategy” opens the way to new questions about the possibility to actually exploit the pharmacogenetic approach to identify predictors of benefit from BV. We believe that future directions in this field of research should necessarily include more comprehensive approaches, to provide an extensive overview of the whole genome. A genome wide association (GWAS) study is currently ongoing, to assess the correlation of genetic profiles with clinical outcome in a wide population of mCRC patients receiving upfront chemotherapy plus BV. A wide biostatistical program is also planned in order to implement the interpretation of data coming from the high number of investigated SNPs in this exploratory GWAS analysis. In particular, adequate statistical tools, such as the multifactor dimensionality reduction (MDR) and the classification and regression tree (CRT) analyses will be applied to accurately estimate and identify gene-gene interactions as previously described [22]–[23]. Two independent cohorts of patients randomized to receive or not the antiangiogenic will serve as validation sets to verify the predictive impact of identified profiles.

In spite of disappointing results reported in this field, the challenge of identifying predictors of benefit from antiangiogenic drugs still represents a hot topic, with increasing consequences on clinical practice. In fact, two phase III randomized trials, ML18147 [24] and BEBYP [25], have recently demonstrated the efficacy of prosecuting BV beyond disease progression in mCRC patients, thus making this field even more complicated. These achievements open the way to new questions about the meaning of traditional clinical progression and its biologic mechanisms while underlining the need for biomarkers of acquired resistance to BV.

## Supporting Information

Table S1 **Multivariable Cox regression models for PFS and OS.**
(DOCX)
