# Supplementary material for: Prospective Validation of Candidate SNPs of VEGF/VEGFR Pathway in Metastatic Colorectal Cancer Patients Treated with First-Line FOLFIRI Plus Bevacizumab
Source: PLoS One. 2013 Jul 4;8(7):e66774. doi: 10.1371/journal.pone.0066774 (PMC3701556; doi:10.1371/journal.pone.0066774)
Supplement: Table S1 — Multivariable Cox regression models for PFS and OS. (DOCX) [file pone.0066774.s001.docx]

**Table S1.** Multivariable Cox regression models for PFS and OS

| **Progression Free Survival** | | | | |
| --- | --- | --- | --- | --- |
| **Characteristics** | | **Adjusted HR** | **95% CI** | ***P**** |
| Mucinous Histology | No | 1 |  |  |
|  | Yes | 1.307 | 0.919, 1.861 | 0.14 |
|  | NA | 1.766 | 1.267, 2.461 | 0.0008 |
| ECOG PS | 0 | 1 |  |  |
|  | 1-2 | 1.508 | 1.095, 2.076 | 0.012 |
| Baseline High LDH | No | 1 |  |  |
|  | Yes | 1.330 | 1.026, 1.725 | 0.031 |
|  | NA | 1.585 | 1.067, 2.354 | 0.023 |
| Primary tumor site | Right colon | 1 |  |  |
|  | Left colon | 0.596 | 0.444, 0.800 | 0.0006 |
|  | Rectum | 0.749 | 0.551, 1.019 | 0.065 |
| Mts site, n | 1 | 1 |  |  |
|  | >1 | 1.586 | 1.236, 2.035 | 0.0003 |
| *VEGFR2* 12505758 | T/T | 1 |  |  |
|  | C- | 1.405 | 1.082, 1.825 | 0.011 |
| **Overall Survival** | | | | |
| **Characteristics** | | **Adjusted HR** | **95% CI** | ***P**** |
| Mucinous Histology | No | 1 |  |  |
|  | Yes | 1.401 | 0.897, 2.190 | 0.14 |
|  | NA | 1.244 | 0.754, 2.053 | 0.39 |
| ECOG PS | 0 | 1 |  |  |
|  | 1-2 | 2.664 | 1.787, 3.972 | <.0001 |
| Baseline High LDH | No | 1 |  |  |
|  | Yes | 1.264 | 0.893, 1.789 | 0.19 |
|  | NA | 1.566 | 0.927, 2.645 | 0.094 |
| Primary tumor site | Right colon | 1 |  |  |
|  | Left colon | 0.454 | 0.311, 0.662 | <.0001 |
|  | Rectum | 0.519 | 0.349, 0.771 | 0.001 |
| Mts site, n | 1 |  |  |  |
|  | >1 | 1.719 | 1.228, 2.407 | 0.002 |
| *VEGFR2* 12505758 | T/T | 1 |  |  |
|  | C- | 1.299 | 0.911, 1.852 | 0.15 |

** P* value was based on Wald test of multivariate Cox proportional hazards model including all variables in table.
